# Treatment options and survival in real life during the past three decades in patients with chronic myelomonocytic leukemia

**DOI:** 10.1007/s10354-022-00976-5

**Published:** 2022-10-25

**Authors:** Julia Reiser, Klaus Geissler

**Affiliations:** 1 Medical School, Sigmund Freud University, Vienna, Austria; 2 Department of Internal Medicine V with Hematology, Oncology and Palliative Care, Hospital Hietzing, Wolkersbergenstraße 1, 1130 Vienna, Austria

**Keywords:** CMML, Azacitidine, Chemotherapy, Hydroxyurea, CMML, Azacitidin, Chemotherapie, Hydroxyurea

## Abstract

The impact of treatment on the outcome of chronic myelomonocytic leukemia (CMML) patients over a longer period of time and the potential role of predictive factors are not well defined. In a retrospective observational study, we analyzed 168 CMML patients regarding treatment options and survival during the past three decades. The proportion of patients treated with hydroxyurea (HU), intensive chemotherapy, and azacitidine (AZA) was 65/19/0% before 2000, 51/25/32% from 2000–2010, and 36/12/53% after 2010, respectively. Median overall survival (OS) increased from 10 months before 2000 to 23 months thereafter (*p* = 0.021). AZA-treated patients but not patients treated with other treatment options had improved survival as compared to CMML patients without AZA therapy (19 vs. 25 months, *p* = 0.041). When looking at subgroups, the following patient cohorts had a significant survival benefit in association with AZA therapy: patients with Hb > 10 g/dL, patients with monocytosis > 10 G/L, and patients with mutations in RASopathy genes.

## Abstract (de)

Der Einfluss einer Behandlung bei Patienten mit chronischer myelomonozytärer Leukämie (CMML) über einen längeren Beobachtungszeitraum und die Rolle möglicher prädiktiver Faktoren wurde bisher nur unzureichend untersucht. In einer retrospektiven Beobachtungsstudie wurden 168 CMML-Patienten im Hinblick auf Therapieoptionen und Überleben während der letzten 3 Jahrzehnte untersucht. Vor dem Jahr 2000 betrug der Anteil von Patienten, die mit Hydroxyurea, Zytostatika und Azacitidin therapiert wurden, 65/19/0 %, zwischen 2000 und 2010 lag er bei 51/25/32 % und nach 2010 bei 36/12/53 %. Das mediane Überleben stieg von 10 Monaten vor dem Jahr 2000 auf 23 Monate danach (*p* = 0,021). Insgesamt wiesen die Patienten, die mit Azacitidin therapiert wurden, eine längere Überlebenszeit auf als CMML-Patienten ohne Azacitidintherapie (19 vs. 25 Monate; *p* = 0,041). Im Hinblick auf Untergruppen hatten folgende Faktoren einen signifikanten Überlebensvorteil in Verbindung mit einer Azacitidintherapie: Patienten mit Hämoglobinwerten ≥ 10 g/dl, mit Monozytose ≥ 10 G/l und Patienten mit Mutationen in den RAS-Genen.

## Introduction

Chronic myelomonocytic leukemia (CMML) is a rare, genotypically and phenotypically heterogenous hematologic malignancy of elderly people with an intrinsic risk of progression and transformation into secondary AML. With regard to the presence of myeloproliferation, CMML was originally subdivided into myeloproliferative disorder (MP-CMML; white blood cell count [WBC] count > 13 × 10^9^/L) versus myelodysplastic syndrome (MD-CMML; WBC count ≤ 13 × 10^9^/L) by the FAB criteria [1]. Since CMML is characterized by features of both MDS and MPN, the World Health Organization (WHO) classification of 2002 assigned CMML to the mixed category, MDS/MPN [2]. CMML is further subclassified by the WHO into three groups based on blast equivalents (blasts plus promonocytes) in peripheral blood (PB) and bone marrow (BM) as follows: CMML‑0 if PB < 2% and BM < 5% blast equivalents; CMML‑1 if PB 2–4% or BM 5–9% blast equivalents; and CMML‑2 if PB 5–19% or BM 10–19% blast equivalents, and/or Auer rods are present [3]. CMML patients may have a highly variable outcome, suggesting that several factors can determine the course of disease and the causes of death in these patients [4–8]. There are a number of established prognostic parameters that have been incorporated into several prognostic models [9–20].

There are various treatment options for CMML patients, which have changed over time [21–36]. Whereas hydroxyurea (HU) and intensive chemotherapy were used to treat myeloproliferation in the past, hypomethylating agents (HMA) such as azacitidine (AZA) and decitabine (DEC) have been introduced in the new millennium. The impact of treatment on the outcome of CMML patients over a longer period of time and the potential role of predictive factors are not well defined. Using the database of the Austrian Biodatabase for Chronic Myelomonocytic Leukemia (ABCMML), we analyzed 168 CMML patients with available information regarding treatment options and survival during the past three decades [37]. This information from a real-life database could be useful for treatment decisions in clinical practice.

## Patients and methods

### Patients

Recently, we have shown that the ABCMML may be used as a representative and useful real-life data source for biomedical research [37]. In this database, we retrospectively collected epidemiologic, hematologic, biochemical, clinical, immunophenotypic, cytogenetic, molecular, and biologic data of patients with CMML from different centers. Clinical and laboratory routine parameters were obtained from patient records. A detailed central manual retrospective chart review was carried out to ensure data quality before analysis of data from institutions. Data curation included the extraction of discrete data elements from patient records, a check for accuracy and consistency of data, and a verification that baseline data were reflective of CMML that was strictly defined according to the WHO criteria [2, 3]. In 168 CMML patients collected from 1.1.1990 until 31.3.2019, information was available regarding treatment options and survival. This research was approved by the ethics committee of the City of Vienna on 10 June 2015 (ethic code: 15-059-VK).

### Molecular studies

Genomic DNA was isolated from mononuclear cell (MNC) fractions of blood samples according to standard procedures. The mutational status of CMML-related protein coding genes was determined by targeted amplicon sequencing using the MiSeq platform (Illumina, San Diego, CA, USA). Details regarding gene panel, library preparation, and data processing have been reported previously [37]. Only variants with an allelic frequency (VAF) ≥ 5%, a described population frequency (MAF) < 1%, and an annotated pathogenic effect (or probability > 90% of being pathogenic), with pathogenicity determined according to public databases and published studies, were used for statistical analysis regarding a potential predictive value for various treatment options.

### Statistical analysis

The log-rank test was used to determine whether individual parameters were associated with overall survival (OS). OS was defined as the time from sampling to death (uncensored) or last follow-up (censored). Dichotomous variables were compared between different groups with the use of the chi-square test. The Mann–Whitney U test was used to compare two unmatched groups when continuous variables were nonnormally distributed. Results were considered significant at *p* < 0.05. Statistical analyses were performed with SPSS v. 27 (IBM Corp., Armonk, NY, USA); the reported *p*-values were two sided.

## Results

### Characteristics of patients

The baseline characteristics of the 168 patients with CMML are shown in Table 1. In order to make comparisons with other published CMML cohorts possible, the percentages of patients regarding established prognostic parameters are given [16, 37]. As seen in other CMML series, there was a male predominance among study patients and more than half of the patients were aged 70 years or older [16]. Interestingly, more than 60% of patients had leukocytosis > 13 G/L, indicating that there was a preference to refer patients with MP-CMML to specialized centers, since this group is below 50% in most reported cohorts.

**Table 1:** Characteristics of chronic myelomonocytic leukemia patients

	Cases *N* = 168	Percent
*Age* *Evaluable* *=* *168*		
< 70 years	63	37.5
≥ 70 years	105	62.5
*Sex* *Evaluable* *=* *168*		
Male	115	68
Female	53	32
*Leukocytes* *Evaluable* *=* *168*		
> 13 G/L	104	62
≤ 13 G/L	64	38
*Hemoglobin* *Evaluable* *=* *168*		
< 10 g/dL	62	37
≥ 10 g/dL	106	63
*Platelets* *Evaluable* *=* *168*		
< 100 G/L	82	49
≥ 100 G/L	84	51
*PB blasts* *Evaluable* *=* *135*		
< 2%	97	72
2–4%	23	17
5–19%	10	7
> 20%	5	4

### Treatment options in different time periods

**Fig. 1:**
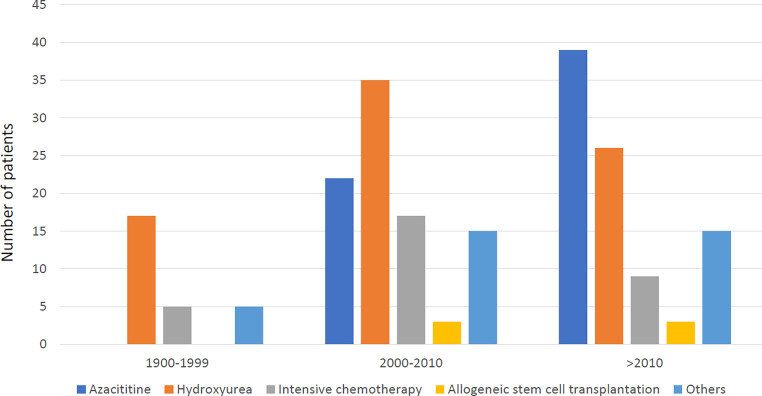
Treatment options for chronic myelomonocytic leukemia patients during different decades

Table 2 shows all the various therapies documented in the ABCMML in at least 5 patients. Accordingly, we formed five treatment groups: hydroxyurea (HU), intensive chemotherapy (ICT), azacitidine (AZA), allogeneic stem cell transplantation, and others. Fig. 1 shows the number of patients receiving these therapies in the different time periods. No therapy information was available before 1990. Between 1990 and 1999, 17/26 (65.4%) patients were treated with HU, 5/26 (19.2%) with AML chemotherapy, and 5/26 (19.2%) with others. AZA was not approved at this time. Between 2000 and 2010, 35/69 (50.7%) were treated with HU, 22/69 (31.9%) with AZA, 18/69 (26.0%) with AML chemotherapy, 15/69 (21.7%) with others, and 3/69 (4.3%) received stem cell transplantation. After 2010, 39/73 (53.4%) were treated with AZA, 26/73 (35.6%) with HU, 9/73 (12.3%) with AML chemotherapy, 15/73 (20.5%) with others, and 3/73 (4.1%) received an allogeneic stem cell transplantation.

**Table 2:** Treatment options in chronic myelomonocytic leukemia patients

Therapy in at least 5 patients	Number of patients who received therapy
Hydroxyurea	78
Azacitidine	61
Intensive AML chemotherapy	19
Lenalidomide	12
Low-dose cytarabine	11
Allogeneic stem cell transplantation	6
Etoposide	5
IL-10	5

*AML* acute myeloid leukemia, *IL-10* interleukin-10

### Survival before and after 2000

**Fig. 2:**
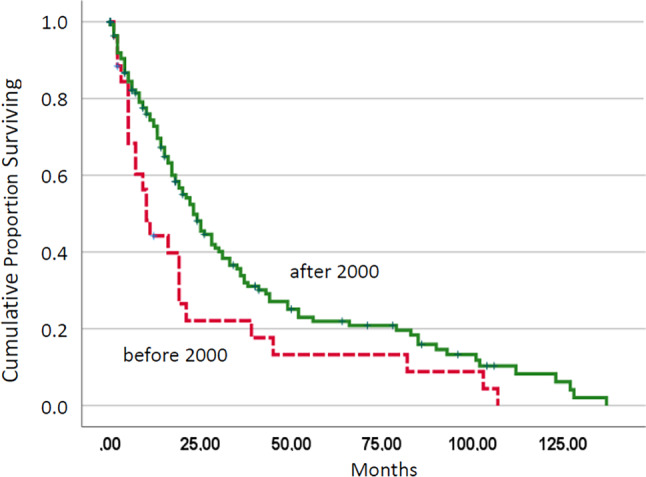
Kaplan–Meier plots for overall survival in chronic myelomonocytic leukemia patients treated before or after 2000

As shown in Fig. 2, there was a significant difference regarding OS between patients treated before 2000 and patients treated thereafter. The median OS before 2000 was 10 months and 23 months in patients in the new millennium (*p* = 0.021). There was no significant difference in survival among CMML patients treated between 2000 and 2010 and patients treated after 2010 (*p* = 0.220). In order to investigate whether the better survival after 2000 may be explained by a disbalance in prognostic factors, we compared established prognostic factors in patients treated before and after January 1, 2000 (Table 3). As one can see, there were no differences regarding these factors, including leukocytosis > 13 G/L, anemia < 10 g/dL, thrombocytopenia < 100 G/L, and the presence of blast cells in peripheral blood. When looking at the changes in median survival in different prognostic groups according to the Mayo score, it was found that numbers were higher after 2000 in all subgroups (median survival in months: 66 vs. 39 in low-risk patients, 35 vs. 9 in intermediate-risk patients, and 15 vs 7 in high-risk patients, respectively).

**Table 3:** Established prognostic parameters in chronic myelomonocytic leukemia patients before and after 2000

	Before 2000 (*n* = 26)	After 2000 (*n* = 142)	*P*-value
Leukocytes G/L; median (range) Evaluable = 167	20.3 (2.8–181)	16.9 (2.5–271)	0.164
Hemoglobin g/dL; median (range) Evaluable = 168	10.4 (7–13.5)	10.8 (4.8–16.5)	0.465
Platelets G/L; median (range) Evaluable = 167	100 (13–303)	98 (1–705)	0.843
PB blasts %; median (range) Evaluable = 135	0 (0–60)	0 (0–69)	0.947

### Survival and different treatment options

**Fig. 3:**
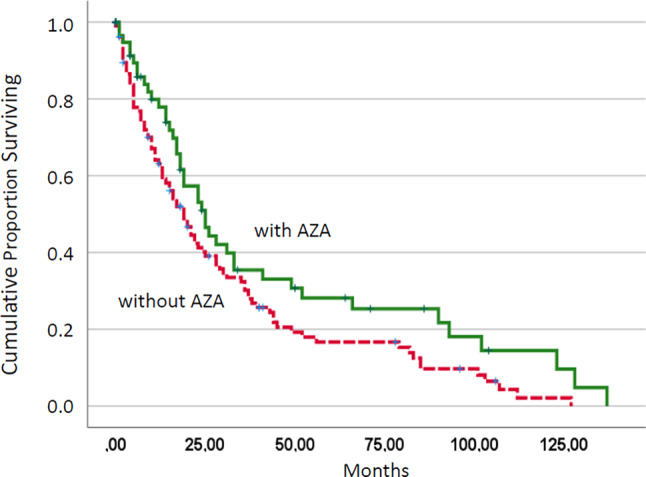
Kaplan–Meier plots for overall survival in chronic myelomonocytic leukemia patients treated with or without azacitidine (AZA)

Table 4 shows the median survival of patients receiving or not receiving specific CMML-directed treatments. It needs to be mentioned that several patients received more than one treatment option, such as HU and AZA. These patients were included in the with and without treatment comparison regarding AZA and HU, respectively. AZA-treated patients but not patients treated with the other treatment options had a significantly improved survival as compared to CMML patients without AZA therapy (19 vs. 25 months, *p* = 0.041; Fig. 3).

**Table 4:** Median survival in chronic myelomonocytic leukemia patients treated with or without various treatment options

	With treatment	Without treatment	*P*-value
Hydroxyurea	19 months	24 months	0.061
Intensive chemotherapy	19 months	22 months	0.301
Azacitidine	25 months	19 months	0.042
Others	33 months	19 months	0.330

### Predictive factors for azacitidine treatment

Since AZA was the only therapy with a significant survival benefit, the predictive value of several factors was examined for AZA. When looking at Table 5, the following patient cohorts had a significant survival benefit in association with AZA therapy: patients with Hb > 10 g/dL, patients with monocytosis > 10 G/L, and patients with mutations in RASopathy genes including *NRAS, KRAS, NF1, PTPN11*, and *CBL*.

**Table 5:** Median survival of chronic myelomonocytic leukemia patients treated with or without azacitidine in various subgroups

	With AZA	Without AZA	*P*-value
WBC < 13 G/L	26 months	29 months	0.174
WBC ≥ 13 G/L	23 months	15 months	0.331
Hb < 10 g/dL	17 months	14 months	0.217
Hb ≥ 10 g/dL	33 months	21 months	0.005
PLT < 100 G/L	23 months	11 months	0.069
PLT ≥ 100 G/L	28 months	31 months	0.099
AMC < 10 G/L	25 months	28 months	0.124
AMC ≥ 10 G/L	24 months	8 months	0.000
PB blasts absent	26 months	21 months	0.092
PB blasts present	19 months	13 months	0.473
*RAS* mutations absent	18 months	36 months	0.863
*RAS* mutations present	25 months	15 months	0.034

*WBC* white blood cell count, *Hb* hemoglobin, *PLT* platelets, *AMC* absolute monocyte count, *PB* peripheral blood, *AZA* azacitidine

## Discussion

The spectrum of treatment options for patients with CMML is continuously expanding. Early reports suggested that etoposide could give good results in CMML, with true complete responses in some cases and improvement rather than worsening of cytopenia [22]. In a randomized phase III trial in patients with proliferative CMML, HU was more effective and achieved a faster response than cytotoxic chemotherapy with VP16 [23]. Interestingly, this study remains the only randomized trial in a pure CMML patient population which demonstrated a survival benefit. Allogeneic stem cell transplantation, which is the only curative therapy, is rarely feasible because of age and/or comorbidities. While unresponsiveness to aggressive chemotherapy is a characteristic for most CMML patients, there may be subgroups that might benefit from more intensive chemotherapy. It is important to note that the approval of hypomethylating agents such as AZA and decitabine (DEC) was originally based on myelodysplastic syndrome studies which included only few patients with CMML. In a phase III clinical multicenter trial of 358 MDS patients including only 11 patients with dysplastic CMML, the median overall survival was 24.5 months in the AZA group as compared to 15.0 months in the conventional care group, leading to the FDA approval of AZA for this subtype of CMML [24]. The approval of DEC for CMML was also based on a phase III clinical trial of 170 patients with MDS, 14 of them with CMML [25]. The ORR was significantly higher in the DEC group versus supportive care (17% vs. 0%, *p* < 0.001), but the median OS was not significantly different between the two arms. Additional phase II studies confirmed the efficacy of hypomethylating agents in all subtypes of CMML and, therefore, these agents are commonly considered as standard of care for higher risk CMML [26–33]. In the largest retrospective study with a pure CMML cohort, patients were treated with AZA (*n* = 56) and DEC (*n* = 65) [34]. The ORRs were 41% according to the IWG MDS/MPN response criteria (AZA-56%, DEC-58%), with CR rates of < 20% for both agents. No significant differences in response rates were seen between MP-CMML and MD-CMML. Similar findings were reported in a smaller prospective phase II Italian study, with 43 CMML patients receiving DEC [33]. The ORR after six cycles was 47.6%, with seven CRs (16.6%), eight marrow responses (19%), one partial response (2.4%), and four hematological improvements (9.5%). After a median follow-up of 51.5 months, median OS was 17 months, with responders having a significantly longer survival than nonresponders. A recent European multicenter randomized phase III trial evaluating DEC ± HU versus HU in advanced MP-CMML did not show significant differences in outcome [35]. Thus, despite these numerous studies, the impact of treatment on the outcome of CMML patients is not well defined. In this retrospective observational study, we show that the outcome of patients improved after the year 2000 and that patients receiving AZA had a better survival that patients not receiving this treatment. Our results are in line with a recent multicenter retrospective study including 949 unselected consecutive CMML patients which investigated whether HMA provides a benefit in subgroups of CMML patients [36]. Adjusted median OS for patients treated with HU versus HMA was 15.6 months as compared to 20.7 months (*p* = 0.0002). In patients with MP-CMML, median OS was 12.6 months as compared to 17.6 months (*p* = 0.0027) for patients treated with HU versus HMA. HMA were not associated with an OS advantage for patients classified as having lower-risk disease (i.e., MD-CMML with < 10% blasts, CMML‑0, or lower-risk CPSS). Considering all the caveats of a retrospective nonrandomized trial, these data suggest HMA as the preferred treatment for patients with higher-risk CMML and those with MP-CMML. Regarding prognosis, a similar observation has been made by the Duesseldorf MDS registry, which reported a change in the prognosis of patients with myelodysplastic syndromes during the past 30 years, with an improvement of survival in patients diagnosed after 2002 (30 vs. 23 months, *p* < 0.0001) [38].

A sometimes impressive but greatly variable response to HMA provides the rationale for searching for biomarkers that predict response. Differentially methylated regions of DNA have been shown to separate DEC responders from nonresponders by Meldi [39]. Other predictors for response to HMA treatment were reported by Duchmann et al. [40]. In a retrospective analysis of 174 CMML patients treated with HMA multivariate analysis showed that mutations in *ASXL1* predicted lower ORR, and *RUNX1* mutations and *CBL* mutations predicted inferior OS. The combination of *TET2* mutation and *ASXL1* wildtype predicted higher CR and better OS. In this study, we also looked at the predictive value of some parameters, including blood picture abnormalities as well as molecular features.

When looking at subgroups, the following patient cohorts had a significant survival benefit in association with AZA therapy: patients with Hb > 10 g/dL, patients with monocytosis > 10 G/L, and patients with mutations in RASopathy genes.

We are aware of the limitations of our study. For example, most of the information used in this study was derived from retrospective real-world data that were not collected systematically or prospectively. Thus, not every parameter was available in all patients. In addition, data from patient records were obtained over many years and from many different centers. Moreover, the patients included in this study were a relatively heterogenous population regarding the blast cell count, and there was a lack of molecular data in a significant number of patients. However, real-world data have recently been recognized as an important way to get insights into the routine management and natural history of rare diseases [41]. CMML is a rare disease and adequate patient numbers for a systematic and prospective study are not easy to collect within a limited timeframe.

In conclusion, the results of our population-based study show an improved survival of CMML patients with the introduction of AZA treatment. Patients without significant anemia and myelomonocytosis due to hyperactivation of the RAS pathway seem to benefit most from this treatment.
